# Comparative study utilizing different post-breeding treatment regimens in cyclic Arabian mares

**DOI:** 10.14202/vetworld.2021.2863-2868

**Published:** 2021-11-10

**Authors:** Khalid Mohammed Karam, Ahmed Saed Alebady, Haitham O. Alhilfi, Dhia Hussain Al-Delemi

**Affiliations:** Department of Surgery and Obstetrics, College of Veterinary Medicine, University of Al-Qadisiyah, Al-Qadisiyah, Iraq

**Keywords:** endometritis, lavage, mare, oxytocin, persistent breeding-induced endometritis, post-breeding

## Abstract

**Background and Aim:**

Post-breeding treatment is the most common practice in the reproductive management of mares. Oxytocin, uterine lavage, and intrauterine (I/U) antibiotic are usually used as prophylactic therapy. This study aimed to determine the most efficient prophylactic treatment regimen among six treatment protocols applied during natural breeding of cyclic Arabian mares.

**Materials and Methods:**

The current study was conducted on cyclic Arabian mares that were subdivided into three age categories (n=968): Category I (5-10 years, n=380), Category II (11-15 years, n=361), and Category III (≥16 years, n=227). Six prophylactic treatments were applied after 4 h of breeding. According to the treatment regimen, treated mares (n=483) were divided into six treatment groups: A (n=80), treated with I/U antibiotic (1 g gentamicin); B (n=81), I/U lavage (normal saline 500 mL); C (n=83), intramuscular (I/M) oxytocin (10 IU); D (n=82), I/U antibiotic and I/M oxytocin; E (n=78), I/U lavage and I/M oxytocin; and F (n=79), I/U lavage with I/U antibiotic and I/M oxytocin. Non-treated mares were classified as controls (n=485). Ultrasonography was performed to monitor pregnant mares 30 and 60 days after mating, and mares were followed until foaling.

**Results:**

Pregnancy and foaling results reveals that in age Category I, treatment with oxytocin alone or oxytocin with I/U lavage showed the highest pregnancy and foaling rates (p<0.01). In age Category II, the highest pregnancy and foaling rates were observed in lavage treatment (p<0.01), whereas, in age Category III, the good pregnancy and foaling rates were monitored in treatment with oxytocin and I/U lavage (p<0.01).

**Conclusion:**

Treatment with systemic I/M oxytocin is ideal in early age group mares (5-10 years of age). However, irrespective of the age categories, all mares exhibited high pregnancy and foaling rates after treatment with systemic I/M oxytocin and I/U lavage with normal saline (0.9%) 4 h post-breeding.

## Introduction

Bacteria and excessive amounts of sperms normally invade the endometrium of mares during natural breeding or insemination [1]. As a normal physical process, fertile mares resolve this problem by pushing out bacteria and excessive spermatozoa from their endometrium through the vagina and vulva at 48 h after breeding. If mares fail in clearing the endometrium beyond the first 48 h, a pathological condition called persistent breeding-induced endometritis (PBIE) develops [2]. PBIE, if untreated, may develop into infectious endometritis or endometrial fibrosis [3].

Infertility in mares due to endometritis is a major problem in equine breeds [4,5], and clinically, mares can be classified according to their resistance or susceptibility to develop PBIE [6,7]. Mares who fail to eliminate inflammation and develop PBIE are mainly older in age [8] or may have problems in vulvar shape or have pendulous uterus [9]. Approximately 10-15% of mares are susceptible to the development of PBIE [10].

Probable PBIE cases are diagnosed clinically by ultrasonography if there is more than 2 cm of uterine fluid in the uterine lumen during estrus [11] or within 36 h after breeding [4]. Treatment after breeding is crucial in decreasing the risk of endometritis and increasing the conception rates [12-14].

There are several treatment protocols used in the field to reduce endometrial inflammation and increase conception rates. This study aimed to compare six treatment protocols used during natural breeding of cyclic Arabian mares with different ages to detect the credible efficient protocol.

## Materials and Methods

### Ethical approval

This study followed the guidelines of the Ethics Committee and current legislation on research and ethical approval of the Faculty of Veterinary Medicine (approval no. VCU-026-2-14), Cairo University, Egypt.

### Study period and location

The study was conducted from May 2012 to January 2015. The study was conducted at Animal Production Section of Countryside Experimental Station at the Research Institute and Private clinics in Cairo Governorate, Egypt.

### Animal

The current study was conducted on healthy cyclic Arabian mares (n=968) with body score condition of 4 and 5 according to Henneke *et al*.’s [15] scoring. Mares were managed in stables in the countryside of Cairo, Egypt, and fed with supplemented balanced ration and checked from May 2012 to January 2015 by veterinary visits. Mares were categorized into three age groups: Category I (5-10 years, n=380), Category II (11-15 years, n=361), and Category III (≥16 years, n=227).

### Post-breeding treatment

The current study was conducted on cyclic Arabian mares that were subdivided into three age categories (n=968): Category I (5-10 years, n=380), Category II (11-15 years, n=361), and Category III (≥16 years, n=227), treated mares (n=483) and were divided intosix groups: A (n=80), treated with intrauterine (I/U) antibiotic (1 g of gentamicin); B (n=81), I/U lavage (normal saline 500 mL); C (n=83), intramuscular (I/M) oxytocin (10 IU); D (n=82), I/U antibiotic and I/M oxytocin; E (n=78), I/U lavage and I/M oxytocin; and F (n=79), I/U lavage with I/U antibiotic and I/M oxytocin. Non-treated mares were classified as controls (n=485). All mares were inseminated by natural mating, and all treatments were applied 4 h post-breeding.

### Ultrasound examination

Pregnancy was confirmed through ultrasonography using B-mode ultrasound attached to a linear probe and at 10 MHz frequency (eSaote, MyLab^®^, Genoa, Italy). Pregnancy was evaluated 30 and 60 days after mating by monitoring embryonic vesicle and embryo, and pregnant mares were followed until foaling.

### Statistical analysis

The entire statistical analysis was conducted using Statistical Package for the Social Sciences version 23.0 (IBM Corp., New York, USA) p<0.05 and 0.01 were used to determine the level of significance. Data were first checked for normal distribution; then, the difference between the treated groups within different age categories was analyzed using Chi-square method.

## Results

All treated cases (n=483) were compared to those that did not receive treatment (control group n=485).

### Day 30 of pregnancy

**Figure-1:**
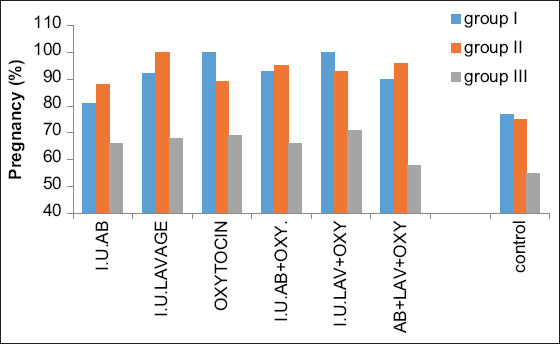
Histogram shows pregnancy 30 days post-breeding in different treatment regimens and in different age category groups.

Pregnancy 30 days after breeding in accordance with the age categories and treatment protocol is presented in Table-1 and Figure-1. In Category I (5-10 years), pregnancy assessment revealed that treatments C and E had the highest pregnancy rate, reaching 100% (p<0.01), while it was 93.47%, 92.10%, and 90.90% for treatments D, B, and F, respectively (p<0.05). Treatment A showed no significant difference (81.48% vs. 77.84%).

**Table-1:** Percentage of pregnancy 30 days post-breeding in different treatment regimens and in different age category groups.

Age categories	Treatments						

	A (n=80)	B (n=81)	C (n=83)	D (n=82)	E (n=78)	F (n=79)	Control (n=485)
I	^c^81.48%	^B^92.1%	^A^100%	^B^93.47%	^A^100%	^B^90.9%	^c^77.84%
II	^B^88.88%	^A^100%	^B^89.74%	^A^95.23%	^A^93.93%	^A^96%	^c^75.25%
III	^B^66.66%	^B^68.75%	^B^69.23%	^B^66.66%	^A^71.42%	^c^58.82%	^c^55.85%

A refers to significant difference (p<0.01) within rows, B refers to significant difference (p<0.05) within rows, C refers to no significant difference within rows

The results of pregnancy assessment in Category II (11-15 years) showed that pregnancy rates for treatments B, F, D, and E were 100%, 96%, 95.23%, and 93.93%, respectively (p<0.01), while those for treatments C and A were 89.74% and 88.88%, respectively (p<0.05), compared to controls (75.25%).

The pregnancy assessment revealed that in Category III (≥16 years), treatment E had the highest pregnancy rate (71.42%) (p<0.01), whereas, in treatments C, B, A, and D, the pregnancy rates were 69.23%, 68.75%, 66.66%, and 66.66%, respectively (p<0.05). However, treatment F showed no difference (58.82% vs. 55.85%).

### Day 60 pregnancy

**Figure-2:**
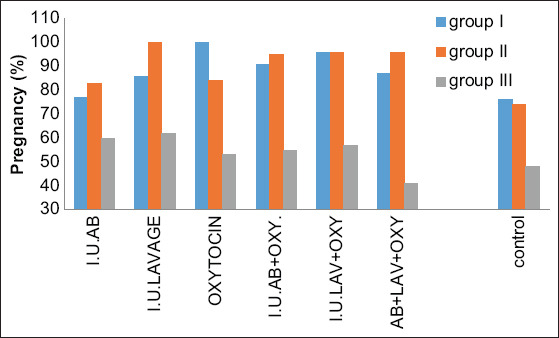
Histogram shows pregnancy 60 days post-breeding in different treatment regimens and in different age category groups.

On day 60 post-breeding, the pregnancy assessment was performed, and the results are shown in Table-2 and Figure-2. The pregnancy assessment in Category I (5-10 years) showed that treatments C and E had the highest pregnancy rates, reaching 100% and 96.42%, respectively (p>0.01), whereas pregnancy rates in treatments D, F, and B were 91.30%, 87.87%, and 86.84%, respectively (p>0.05). However, the pregnancy rate in treatment A was not different (77.77% vs. 76.62%).

**Table-2:** Percentage of pregnancy 60 days post-breeding in different treatment regimens and in different age category groups.

Age categories	Treatment						

	A (n=80)	B (n=81)	C (n=83)	D (n=82)	E (n=78)	F (n=79)	Control (n=485)
I	^c^77.77%	^B^86.84%	^A^100%	^B^91.3%	^A^96.42%	^B^87.87%	^c^76.62%
II	^B^83.33%	^A^100%	^B^84.61%	^A^95.23%	^A^93.54%	^A^96%	^c^74.43%
III	^B^60%	^A^62.25%	^c^53.84%	^c^55.55%	^B^57.14%	^c^41.17%	^c^48.44%

A refers to significant difference (p<0.01) within rows, B refers to significant difference (p<0.05) within rows, C refers to no significant difference within rows

The pregnancy assessment in Category II (11-15 years) revealed that pregnancy rates in treatments B, F, D, and E were 100%, 96%, 95.23%, and 93.54%, respectively (p<0.01). Pregnancy rates in treatments C and A were 84.61% and 83.33%, respectively (p<0.05).

The pregnancy assessment in Category III (≥16 years) showed that treatment B has the highest results, reaching 62.25% (p<0.01), whereas rates in treatments A and E were 60% and 57.14%, respectively (p<0.05). However, treatments D, C, and F did not show a significant difference in pregnancy rates compared to controls (55.55%, 53.84%, and 41.17% vs. 48.44%).

### Foaling day

**Figure-3:**
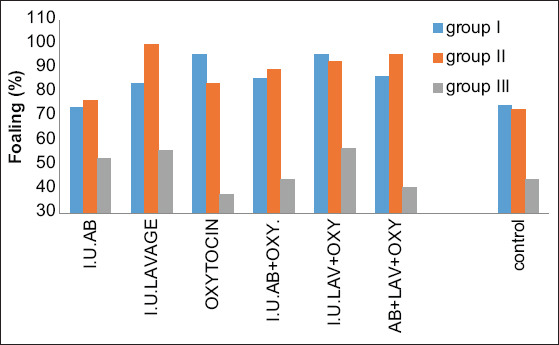
Histogram shows foaling in different treatment regimens and in different age category groups.

All pregnant mares were followed until foaling; the foaling percentage is shown in Table-3 and Figure-3. In Category I, the highest foaling percentages were noted in treatments C and E (96.87% and 96.42%, respectively) (p>0.01), and percentages in treatments F, D, and B were 87.87%, 86.95%, and 84.21%, respectively (p>0.05). However, treatment A showed no significant difference (74.07% vs. 75.56%).

**Table-3:** Percentage of foaling in different treatment regimens and in different age category groups.

Age categories	Treatment						

	A (n=80)	B (n=81)	C (n=83)	D (n=82)	E (n=78)	F (n=79)	Control (n=485)
I	^c^74.07%	^B^84.21%	^A^96.87%	^B^86.95%	^A^96.42%	^B^87.87%	^c^75.56%
II	^c^77.77%	^A^100%	^B^84.61%	^B^90.47%	^A^93.54%	^A^96%	^c^73.23%
III	^B^53.33%	^B^56.25%	^c^38.46%	^c^44.44%	^B^57.14%	^c^41.17%	^c^44.14%

A refers to significant difference (p<0.01) within rows, B refers to significant difference (p<0.05) within rows, C refers to no significant difference within rows

In Category II, the highest foaling rates were observed in treatments B, F, and E (100%, 96%, and 93.54%, respectively, p>0.01) and treatments D (90.47%) and C (84.61) (p>0.05), whereas treatment A showed no significant difference (77.77% vs. 73.23%).

In Category III, the rates in treatments E, B, and A were 57.14%, 56.25%, and 53.33%, respectively (p>0.05), whereas treatments D, F, and C did not show a significant difference with the controls (44.44%, 41.17%, and 38.46% vs. 44.14%).

## Discussion

Some mares are susceptible to develop endometritis after artificial insemination or natural mating because of considerable disturbances in mares’ defense mechanisms or due to anatomical malformations. However, in certain mares, the defense system is inefficient, and mares become vulnerable to endometritis before spontaneous mating or artificial insemination. Moreover, the uterine fluid has the potential to function as a culture medium for bacteria that may enter the uterus and reduce spermatozoa and subsequently embryo survival [16,17]. Endometritis after insemination or mating is a common problem that usually causes infertility or early embryonic death [17,18]. Persistent endometrial inflammation may develop into chronic infectious endometritis, which is observed in 25-60% of barren mares [19,20], due to the presence of many microorganisms in the uterus, mostly *Escherichia coli* and *Streptococcus zooepidemicus* [4,16,21]. Several treatments, traditional and nontraditional, were used after mating to reduce inflammation and maintain fertility. Traditional treatments, such as uterine lavage, injection of oxytocin, and I/U antibiotic administration, were used after mating in many clinical programs to maintain mares’ fertility [13,14,17,20-22].

The benefits of oxytocin injection after breeding are to stimulate the contractions of uterine muscles to eliminate excessive sperms and bacteria that enter the uterus during natural mating or insemination [23,24]. Oxytocin is widely used during estrus or foal heat because of its contractile action on the myometrium that accelerates uterine involution [25] and mucosal degeneration [26]. The effective oxytocin dose that produces myometrial contractility and enhances mares’ fertility is 10-20 IU [27,28]. Oxytocin can be injected after 4 h of breeding because sperms complete their transport to the oviduct around that time; thus, treatment with oxytocin and uterine lavage can be performed after 4 h post-breeding without any interference with sperm movement and mare fertility or pregnancy rates [29,30].

Uterine lavage by normal saline after breeding has been used repeatedly in farms because of its ability to increase the tone of uterine muscles [31] and its efficiency to clear the uterine lumen from microorganisms, dead sperms, inflammatory cells, and debris, which are known to be possible reasons for early embryonic death [32,33]. Uterine lavage can reintroduce active viable neutrophils that can induce microbial degradation in stagnant inflammations [22].

Gentamicin is an aminoglycoside bactericidal and broad-spectrum antibiotic that is used repeatedly to treat bacterial endometrial infections in mares [34]. Its mechanism of action involves inhibition of bacterial protein synthesis by binding to 30S subunit of bacterial ribosomes; therefore, gentamicin is indicated as one of the broad-spectrum antibiotics for the treatment of acute serious infections, such as those caused by Gram-negative bacteria [35]. In the current study, administration of a single dose of gentamicin as I/U antibiotic has little effect on pregnancy rates in mares compared with other prophylactic regimens. This is similar to the findings that were reported in a previous study [36]. Moreover, they assume that the I/U route may irritate the endometrium and might lead to inconsistent tissue penetration, which facilitates the development of antimicrobial resistance. Administration of antibiotic with uterine lavage in combination with oxytocin injection was reported as a good protocol to increase mares’ fertility [12,13].

Our results revealed that administration of oxytocin alone resulted in significant foaling rate in age Categories I and II but not in Category III. It might be possible that older age mares with multiple foaling histories respond less to oxytocin than younger mares; furthermore, the size of the uterus in these mares might not permit the complete evacuation of semen and debris after mating after treatment with oxytocin only.

According to our results, it seems that I/U lavage by normal saline in combination with oxytocin injection is a common best protocol in all three age groups and showed the highest percentages of pregnancy in all groups with significant differences compared with the controls of the same ages; therefore, we recommend using this protocol after 4 h of every stallion mount in Arabian mare breeding programs.

## Conclusion

To reduce possible post-breeding uterine inflammation cases in cyclic Arabian mares, six treatment protocols were applied on mares at different ages after natural breeding. Uterine lavage + oxytocin injection is the best treatment protocol for all ages of cycling Arabian mares that reduce uterine inflammation after natural mating and increase pregnancy and foaling rates.

## Authors’ Contributions

KMK, ASA, HOA, and DHA: Involved in the conception of the research idea and methodology design, performed the data analysis and interpretation, and prepared the manuscript for publication. KMK and DHA: Participated in the design of the methodology and involved in fieldwork. KMK: Project super­vision, study design, and data interpretation. ASA and HOA: Participated in the manuscript preparation, data analysis, and contributed their scientific advice during the work and revision. All authors read and approved the final manuscript.
